# Achieving national fitness goals through school-community collaboration: a configurational analysis of multi-agent games in China

**DOI:** 10.3389/fpubh.2026.1831733

**Published:** 2026-05-14

**Authors:** Zhuang Zhuang, Pengfei Tai, Zhaojin Li, Fugao Jiang

**Affiliations:** 1 School of Sports Science, Qufu Normal University, Jining, China; 2 Shandong Rural Revitalization Sports Research Institute, Qufu Normal University, Jining, China

**Keywords:** configurational paths, multi-agent game, national fitness, public health, school sports ground, school-community collaboration

## Abstract

**Objective:**

The persistent spatial inequality in the distribution of fitness resources is still the key obstacle to achieve the goal of nationwide sports activities. Although school stadiums and gymnasiums account for more than half of the national sports infrastructure, their potential has not been realized to a large extent due to the decentralized governance among community residents, institutional managers and end users. This division has created a gap in implementation and undermined the cooperation between schools and communities in health equity.

**Methods:**

From the perspective of game theory, the opening of school sports venues is regarded as the result of multi-agent strategic interaction among Social fitness population, School administrators and students. Using the method of fuzzy set qualitative comparative analysis (fsqca), this paper discusses the formation mechanism of School sports resources sharing efficiency.

**Results:**

There is no single necessary condition for the sharing of School sports resources; On the contrary, its implementation depends on the concurrent combination of multi-agent conditions. This study further identified three typical configuration paths: the social driving path led by Social self-organization, cultural identity and common responsibility; School led path supported by system investment, Policy compensation and school society cooperation; Student participation path based on right protection and Security awareness. The robustness check confirms the stability of these paths during calibration and adjustment.

**Conclusion:**

These research results show that the opening of school venues as a complex adaptive system function, in which the stakeholder configuration determines the success. Alternative governance paths provide operational insights for situational policy design in resource constrained environments, and help integrate educational infrastructure into the community health promotion system. The multi-agent cooperation mechanism can eliminate the structural barriers of national fitness participation, so as to promote the implementation of scientific development in the field of public health.

## 1 Introduction

The popularity of national fitness is an important symbol of a country’s modernization (1). The World Health Organization (WHO) released the 2018–2030 physical activity global action plan: more active people, a healthier world, which proposed that “by 2030, the global prevalence of lack of physical activity among adults and adolescents will be reduced by 15%”. The Chinese government issued the opinions on building a higher level public service system for national fitness, which pointed out that optimizing the layout of resource allocation, building a higher level public service system for national fitness, and realizing the equalization of sports public services are important cornerstones for achieving sports power (2). As the core element of the public service system of national fitness, the fairness of space allocation and the accuracy of service supply of sports resources are directly related to the strategic support effectiveness of the “national fitness plan” and the practical achievement of the construction goal of “sports power” (3, 4). According to the Sixth National Sports Ground Survey (2014), the sports ground area of colleges, universities, and primary and secondary schools accounts for 50.76% of the total national sports facility area (5), highlighting the structural dominance of school-based sports resources in China. Under resource-sharing conditions, campus sports venues have increasingly become preferred locations for public physical activity (6). However, recent demographic and urbanization trends reflected in the Seventh National Census and related statistics (2023) indicate significant changes in population distribution, aging structure, and urban demand for public fitness services. This suggests that while the supply structure of sports resources remains relatively stable, the demand side is undergoing rapid transformation. As a result, the divergence between stable supply and evolving demand has intensified the mismatch between sports resource allocation and public fitness needs. In this context, activating existing school sports facilities through resource-sharing mechanisms becomes increasingly critical.

Throughout the use of utilization of school sports resources in developed countries, many countries have greatly promoted the efficient use of school sports resources at the level of institutional guarantee. The sharing mode of school sports resources and facilities led by the Canadian government has not only improved the utilization rate of public resources, but also reduced the cost of repeated construction to a great extent; London, on the other hand, conducts rational planning based on demand, adopts the cross-sectoral cooperation mechanism of investment and co-construction, requires schools to open venues to the community during non-teaching hours, provides free equipment and venues to students and external institutions, and establishes long-term cooperation with grassroots clubs; similar to London, the Danish government uses multiple cooperation among schools, governments and enterprises to build multifunctional communities, which are open to the public during non-teaching hours and become a benchmark for the sustainable development of sports resource sharing (7).

As a typical sample of urbanization and demographic transition in developing countries, the fundamental dilemma of National Fitness Resources lies in the following aspects: ① The contradiction between the rapid development of urban real estate and the spatial justice of fitness resources has become increasingly prominent. In the process of urbanization, driven by land financial interests, space is gradually commercialized, squeezing public sports venues into the peripheral area of urban space reproduction, resulting in the structure of the Capital center gathering and marginalization of health space. This occupation of space rights and interests breaks the accessibility of fitness resources and daily activities of residents; ② School and community sports resources are separated. School venues are “self-enclosed” due to safety responsibility and cost avoidance, while community facilities are “inefficient supply” due to professional operation and maintenance ability, which together constitute a rigid constraint on resource flow; ③ Government guidance is divorced from social needs. The government-guided “technology governance” model relies too much on standardized indicators, turning national fitness into an administrative task of assessment, leading to the alienation between policy design and residents’ fitness needs. Guided by efficiency priority, it squeezed the institutional space for the collaborative participation of multiple subjects, resulting in the failure to realize the paradigm shift from “policy supply led” to “demand response driven.”

Based on the framework of Game theory framework, this paper uses the method of fsQCA to reveal the configuration effect of multi-dimensional element collaborative linkage on School sports resources sharing, so as to deconstruct the complex causal mechanism of Allocation of public sports resources. At the same time, it provides theoretical support and practical guidance for the optimization of the allocation of sports resources, the sharing of sports venues under the background of urban renewal, and the deep integration of intensive use of land resources, which will help promote the high-quality development of national fitness and help realize the Healthy China strategy.

## 2 Literature review and theoretical framework

### 2.1 Literature review

People’s health is an important symbol of Socialist modernization (8). The outline of the “Healthy China 2030” plan requires that people’s health be the center, and everyone should enjoy basic sports and fitness services. At the same time, it clearly points out that “coconstruction and sharing, Health for All” is the theme and basic path of building a Healthy China strategy strategy (9). President Xi Jinping put forward that “without Health for All, there is no comprehensive well-off society.” Physical exercise is an indispensable means to promote the construction of a Healthy China and improve the health level of the People’s Health. At present, there is a significant gap between the construction and utilization of sports venues and facilities in China and the growing demand of the people for sports activities, which not only reflects the structural imbalance between supply and demand of public sports resources, but also highlights the necessity of improving resource allocation efficiency and governance coordination. In order to fundamentally solve this deep-seated contradiction between supply and demand, we must focus on solving the problem of lack of sports venues around the people. Wang (10) made a comparative analysis of the public service system of national fitness between China and the United States, and believed that China’s discussion of Macro objectives and Microscopic objectives was vague, and the specific tasks were not particularly clear, which brought great difficulties to the implementation of later work, suggesting that existing studies tend to focus on policy objectives but still lack sufficient explanation of how these objectives are translated into effective governance mechanisms at the micro level. With the implementation of the “Healthy China 2030” planning outline, the core position of national fitness in the sports power and Healthy China strategy has been emphasized (11). China has entered a period of high-level construction of national fitness. Its function has changed from “understanding the people’s livelihood” to “quality of life”. At the same time, it is also an important part of complying with the inherent requirements of the people’s expectations for a high-quality life and promoting the common prosperity of all people to make significant and substantial progress (12, 13), which further indicates that improving the efficiency and sustainability of sports resource sharing has become a key research issue.

In recent years, the national fitness facilities have been continuously improved, but the lack of public fitness venues is still one of the manifestations of the imbalance in the distribution of social resources. In alleviating the contradiction between the demand for fitness and the lack of public sports facilities, it is pointed out that in addition to increasing the supply of sports facilities, it has become an important choice to further activate the existing resources and speed up the opening of school sports facilities to the public (14). In practice, many countries’ school venues have implemented open policies during non-teaching periods, such as European and American countries’ bringing school sports spaces into public use during non-classroom periods (15), indicating that institutional arrangements can effectively improve the utilization efficiency of public sports resources. However, there are also many challenges in practice: the difficult problem of policy coordination appears, the divergence of interests between schools and communities is an important obstacle to the opening of venues, as well as the imperfect management system for the opening of schools to the society (16), the utilization and loss rate of sports facilities (17), the safety of students and residents (18), and the unclear responsibilities of the government and schools (19). As a result, national policies, local governments, schools and society have been in a state of separation for a long time on the issue of opening school facilities to the public, which needs to be solved as a whole, which reveals that existing studies mainly emphasize institutional constraints and practical dilemmas, but lack in-depth analysis of the interaction and coordination mechanisms among multiple actors involved in resource sharing.

fsQCA has been widely used in social science research, gradually forming research directions represented by public policy implementation (20), health behavior mechanism (21), organizational governance performance (22) and multi-agent collaboration (23). The study believes that fsQCA can effectively reveal the complex causality under the concurrent effect of multiple antecedents, emphasizing the asymmetry of causality and multi-path equivalence, and is especially suitable for explaining the realistic situation of “one result and multiple causes” in public affairs (24). In the fields of health governance, public service supply and public resource allocation, fsQCA is widely used to reveal the multiple paths for different combinations of conditions to achieve high performance results, rather than testing the marginal effect of a single variable (25, 26), demonstrating its advantage in explaining complex governance outcomes beyond traditional linear approaches. As a typical public resource sharing behavior, the operation result of the opening of school sports venues does not depend on a single policy or subject decision, but the comprehensive embodiment of the strategic interaction of multiple subjects under different constraints. There are obvious concurrency and context dependence between different conditions (27, 28). This process is analyzed from the perspective of condition combination and implementation path. The fsQCA method provides a more consistent analysis perspective for systematically revealing the formation mechanism of school sports venue opening performance by identifying the equivalent path under the synergistic effect of multiple conditions (29), but current research has not sufficiently integrated this configurational approach with a multi-actor interaction framework, especially in the specific context of school sports resource sharing.

Compared with existing studies that primarily focus on single-factor explanations or general governance contexts, therefore, based on Game theory, this study introduces fsQCA analysis method, aiming to break through the single factor interpretation paradigm, compare the implementation path of efficient opening of school sports venues under different combinations of conditions, further clarify the collaborative logic of venue opening, reveal the configuration mechanism of multi-agent collaborative governance, and provide a more explanatory and operational empirical basis for the opening and governance optimization of school Open sports resources under the background of national fitness, thereby explicitly integrating multi-actor interaction theory with configurational analysis, and contributing to a more systematic explanation of how multiple conditions jointly shape resource sharing efficiency.

### 2.2 Theoretical framework

The opening of school sports venues to the outside world is a typical public resource sharing decision. Its operation mechanism is not derived from the rational choice of a single subject, but the result of the strategic game formed by the social fitness group, school administrators and students around the resource usage rights, safety responsibility and cost benefit distribution. Under the realistic background that the demand for national fitness continues to grow and the supply of public sports resources is structurally inadequate, school sports venues have gradually changed from closed educational internal resources to quasi public products with public attributes, which inevitably leads to a continuous and complex interaction between multiple subjects (30, 31). From the perspective of the main structure of the game, the Social fitness population, school administrators, and students constitute the core action unit of the opening of school sports venues, but there are significant differences in the risk tolerance and responsibility bearing mode among the three types of subjects, which lays the foundation for the subsequent game conflict and strategy differentiation.

**Figure 1:**
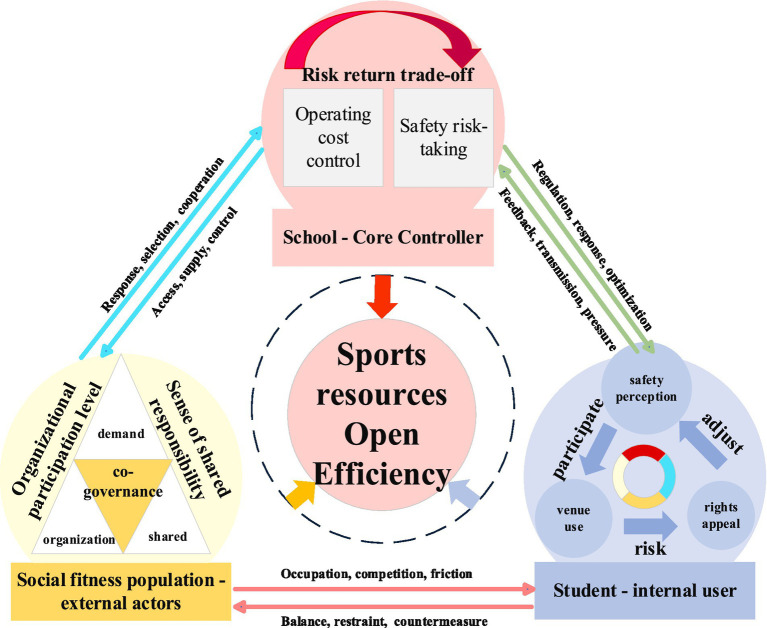
Tripartite game diagram of school sports venues opening.

In the specific game process, as potential users of sports venues, the Social fitness population’s core goal is to improve the availability and convenience of fitness resources, so they generally tend to support a higher level of venue opening in terms of strategy selection. However, since the Social fitness population does not directly bear the main cost of site maintenance and safety management, when the responsibility constraint and sharing mechanism are insufficient, it is easy to induce “free riding” behavior, thus weakening the stability of the cooperation structure (32). From a game-theoretic perspective, the “level of shared responsibility” redistributes payoff structures among actors, thereby aligning individual incentives with collective outcomes and mitigating collective action dilemmas. Higher levels of shared responsibility increase the expected utility of cooperation and discourage opportunistic behavior. Similarly, the “maturity of risk management” reduces uncertainty and perceived risks, lowering the expected costs of opening strategies and facilitating the emergence of stable cooperative equilibria. As the actual owner and governance subject of sports venues, school administrators need to balance public service responsibility, campus safety risks and operating cost control, and their strategy selection usually shows obvious risk aversion characteristics (33, 34). Although students do not directly participate in decision-making, their demands for use rights and security will reverse constrain the decision-making space of school administrators through changes in satisfaction, conflict risks or rising management costs (35). Therefore, students are not treated as independent decision-makers in this study, but rather as constraint-based actors who indirectly shape the decision environment and strategic choices of school administrators. Consequently, there is no optimal solution dominated by a single subject in the opening of school sports venues, but a game equilibrium formed under the mutual checks and balances of multiple subjects (Figure 1) (Question 3.2).

From the perspective of the nature of the game, the opening of school sports venues has the characteristics of both a non-zero sum game and a repeated game. The result is not determined by a one-time decision, but a process of continuous evolution under the conditions of bounded rationality and Incomplete information (36–38). In different situations, the combination differences of Level of shared responsibility of Social fitness population, school risk management ability and students’ security awareness will lead the game structure to converge to different equilibrium states. When multiple conditions form a complementary relationship, collaboration strategy is easier to evolve into a stable state; on the contrary, even if a single condition is favorable, the cooperation may break down due to the lack of other conditions (39–41). The configuration path for its implementation needs to be scientifically analyzed to determine its diversity and equivalence.

Building on the above analysis of equilibrium outcomes in multi-agent interactions, the opening of school sports resources can be understood as the result of strategic interactions among different actors under specific institutional and social conditions. This study therefore focuses on how such interactions are reflected in different combinations of conditions associated with resource sharing efficiency. In this context, fsQCA is employed to identify configurational patterns through which multiple conditions jointly contribute to these outcomes, which can be interpreted as relatively stable states of interaction under given conditions. This approach enables an outcome-oriented analysis of complex multi-agent interactions, while drawing on game theory to interpret the underlying logic of interdependence and mutual constraints among actors.

## 3 Research methods and data construction

### 3.1 Method selection

The results are often determined by the interaction of various explanatory factors (42), while the traditional measurement model is difficult to solve the Configurability problem among multiple factors. FsQCA is an increasingly emerging method in Social Sciences (43), which is the reasoning of separating fuzzy combinations of variables. It uses a Boolean system to provide a new analytical perspective for empirical research to a certain extent (44). This paper examines whether a single antecedent constitutes an indispensable constraint for the opening of school sports venues through the necessity analysis, and further identifies which combinations of antecedents can promote the opening of school sports venues under the Joint effect through the sufficiency relationship, so as to reveal multiple implementation paths corresponding to different strategy configurations.

Thus, fsQCA provides an effective analysis tool for identifying the Configuration features of school sports venues opening, and helps to transform the implicit Strategic Interactive Relationship in the process of multi-agent game into a comparable and verifiable combination of conditions. On this basis, fsQCA restricts the interpretation results of the combination of multivalued conditions through the systematic evaluation of consistency and coverage under the framework of set theory, so as to reduce the conflict between the results in the analysis process, and provide a more robust methodological support for the further implementation of the analysis of the open game mechanism of school sports venues (45).

### 3.2 Data collection

This study uses the Quantitative empirical research paradigm to systematically measure core variables through the Structured scale. Based on the characteristics of cross-group research, the sampling scheme employs a non-probability sampling method, which offers significant advantages in terms of cost-effectiveness, operational convenience, and data quality. The research framework includes three groups: members of the public who engage in fitness activities, school administrators, and students in attendance. According to the differences of each group, a targeted combination of measurement tools is designed.

The research focuses on the cognitive differences and demand characteristics among various stakeholder groups regarding the open policy for school sports resources, and adopts a multi-dimensional measurement approach incorporating a mandatory response mechanism, which mitigates the risk of missing data to some degree. The questionnaire was designed, distributed, collected and processed in accordance with the ethical requirements of the Declaration of Helsinki, and was distributed from March 1, 2025 to June 1, 2025. Among them, 109 valid samples were from members of the public fitness community, 131 valid samples were from school officials, 186 valid samples were from current students, and the total number of valid samples was 426, which met the sample size standard for cross-group comparative analysis. This study has been approved by the Ethics Committee of Qufu Normal University (approval code: 2025161). Participants’ consent has been obtained in the survey.

### 3.3 Variable description

In the research design, the Conditional variable system is constructed from three levels of Social fitness population, School administrators and students, and the Multi agent mechanism of action of Opening up of school sports resources is systematically analyzed. In the school dimension, System investment control, Space time allocation elasticity, Integrity of Open Policy, Policy compensation obtained, Maturity of risk management and Depth of school community collaboration are selected to assess the school’s ability based on technical support, Institutional implementation, risk control and collaborative governance; in the social dimension, Digital Divide System, Degree of spontaneous organization, Penetration of Health Culture, Level of shared responsibility, Fitness demand intensity and Diversity of demand are introduced to analyze the external impact of social technology environment, organization mobilization and demand structure on resource sharing; in the student dimension, around Proficiency in digital platforms, venue use rules, Rights appeal mechanism, Student Autonomous Organization, sports participation frequency and Security Perception Index, students’ differences in technology adaptation, system perception and willingness to participate are reflected, thereby capturing their role as key stakeholders who influence the usage environment and indirectly shape decision-making outcomes.(Question 3.2) Finally, “Resource sharing efficiency” is taken as the outcome variable to comprehensively measure Resource allocation efficiency, Service accessibility and public satisfaction, providing the basis for identifying the multi-path implementation mechanism of Opening up of school sports resources (Table 1).

**Table 1:** Variable description.

Variable type	Variable dimension	Variable name	Variable interpretation
Conditional variable	School dimension	System investment control	Digital infrastructure maintenance and software system iteration are the core of technical capability building (52)
		Space time allocation elasticity	Financial guarantee and process control of technology investment directly affect resource utilization efficiency (53)
		Integrity of Open Policy	The completeness of policies is the basis of “system support,” and the implementation rules determine the feasibility of the resource opening system (54)
		Policy compensation obtained	Policy compensation obtained is the core of “interest balance,” and the scientific nature of the compensation mechanism directly affects the internal driving force of resource openness
		Maturity of risk management	Maturity of risk management can effectively avoid the problem of alienated use of resources and ensure the benign operation of open ecology
		Depth of school-community collaboration	The school has specialized venues, while the community has operational experience. The coordination of the two can break through the dilemma of “resource island” (55)
	Social dimension	Digital divide system	Digital divide technology conforms to the theory of “technological externality,” that is, the technical conditions take into account the balance between the subjects (56)
		Degree of spontaneous organization	The participation degree of community sports cooperation directly reflects the core index of the equalization level of public services
		Penetration of Health Culture	The popularization depth and recognition breadth of health culture determine the mass base of resource opening
		Level of shared responsibility	The level of shared responsibility reflects the key to “collaborative effectiveness” and can effectively resolve collective action dilemmas such as “free riding”
		Fitness demand intensity	The accuracy and urgency of fitness demand orientation determine the targeting of resource supply
		Diversity of demand	Diversity of demand can meet the needs of the whole population and the whole cycle of sports and health, and eliminate the structural contradictions caused by service homogeneity
	Student dimension	Proficiency in digital platforms	Students’ technical literacy is the core variable of the digital network and resource-driven path, which will directly affect resource utilization efficiency (57)
		Site use rules	Institutional constraints on students’ use of time, behavior norms and safety responsibilities in the process of Open sports resources
		Rights appeal mechanism	The formal feedback and appeal system that can be followed when the legitimate rights and interests are affected in the use of sports resources
		Student Autonomous Organization	Participate in the management and maintenance of sports venues in the form of autonomy, so as to realize self-discipline (58)
		Sports participation frequency	High frequency participation is not only the result of internal drive, but also the external incentive of the environmental atmosphere
		Security Perception Index	Students’ trust in the safety of site facilities and management transparency affects their behavior choices (59)
Result variable		Resource sharing efficiency	This variable integrates resource allocation efficiency, service accessibility, public satisfaction and other core elements, and responds to the core research proposition of “improving the utilization efficiency of school sports resources”

### 3.4 Data calibration

Calibration is the process of assigning a case to a set membership (46). Recalibrating a variable into a set requires establishing three threshold values based on theoretical criteria: full membership, crossover point, and no membership. The resulting set membership ranges between 0 and 1 (47). The purpose of calibration is to determine the degree to which a case belongs to a certain set (48). In this paper, 95% quantile, 50% quantile and 5% quantile are selected to correspond to three anchor points (49), namely, full membership, intersection and complete non membership (Table 2). The variables exhibit sufficient variation across cases, making percentile-based calibration appropriate for distinguishing different levels of membership. This approach enhances the comparability of cases and ensures the robustness of the results.

**Table 2:** Calibration of antecedent and result conditions.

Social fitness participants				School administrators				Student			
Variable name	Full subordination	Intersection	Completely unsubordinated		Full subordination	Intersection	Completely unsubordinated		Full subordination	Intersection	Completely unsubordinated
Variable name	0.95	0.5	0.05	Variable name	0.95	0.5	0.05	Variable name	0.95	0.5	0.05
Fitness demand intensity	3	2	1	Integrity of Open Policy	2.667	2	1	Sports participation frequency	3.333	2	1
Diversity of demand	3	2	1	System investment control	2	1.2	1	Site use rules	3.25	1.75	1
Digital divide coefficient	2.6	2	1.2	Policy compensation obtained	4	2.25	1	Rights appeal mechanism	3	2	1
Degree of spontaneous organization	3.2	2.3	1	Maturity of risk management	3.5	2.25	1	Student Autonomous Organization	3.2	1.8	1
Penetration rate of health culture	3.6	2.3	1	Space–time allocation elasticity	3.166	2.333	1	Proficiency in digital platforms	2.8	2	1
Level of shared responsibility	3.75	2.5	1	Depth of school community collaboration	3	2.2	1	Security Perception Index	3.333	2.333	1
Resource sharing efficiency	3	1.667	1	Resource sharing efficiency	2.75	1.75	1	Resource sharing efficiency	2.8	2	1

Through the selection of anchor points, this paper conducts data calibration for each group separately, focusing on the questionnaires of fitness enthusiasts from the community, school administrators, and students. After calibration, the membership value of the fuzzy set in some cases is exactly 0.5, potentially disrupting classification and resulting in inaccurate outcomes. To solve this problem, add 0.001 to the calibrated value of 0.5.

## 4 Analysis of empirical results

### 4.1 Antecedent condition necessity analysis

Necessary condition analysis involves ascertaining whether there exists a single factor that independently influences the result by examining whether a single antecedent condition constitutes a necessary condition for the result. When the coherence reaches 0.9, this variable is considered a prerequisite for achieving the result (50). In order to ensure the robustness of the results, the study also tested the univariate non high results, and the results showed that the consistency of all conditions was less than 0.9. (Table 3) indicates that there is no necessary condition leading to the sharing and opening of school sports resources. Given that these values remain sufficiently below the conventional threshold, minor adjustments to the cutoff would not alter the substantive conclusion. This suggests that the identification of no necessary condition is robust and not sensitive to reasonable variations in the threshold setting.

**Table 3:** Necessity analysis of individual factors.

Social fitness population			School administrators			Student		
Variable name	Uniformity	Coverage	Variable name	Uniformity	Coverage	Variable name	Uniformity	Coverage
Fitness demand intensity	0.561	0.706	Integrity of Open Policy	0.731	0.610	Sports participation frequency	0.672	0.621
~Fitness demand intensity	0.588	0.558	~Integrity of Open Policy	0.557	0.488	~Sports participation frequency	0.599	0.628
Diversity of demand	0.631	0.738	System investment control	0.713	0.699	Site use rules	0.539	0.624
~Diversity of demand	0.614	0.547	~System investment control	0.573	0.434	~Site use rules	0.688	0.588
Digital divide coefficient	0.676	0.704	Policy compensation obtained	0.635	0.567	Rights appeal mechanism	0.645	0.721
~Digital divide coefficient	0.578	0.569	~Policy compensation obtained	0.634	0.519	Rights appeal mechanism	0.629	0.551
Degree of spontaneous organization	0.809	0.822	Maturity of risk management	0.646	0.535	Student Autonomous Organization	0.707	0.684
Degree of spontaneous organization	0.438	0.441	~Maturity of risk management	0.598	0.528	Student Autonomous Organization	0.555	0.554
Penetration rate of health culture	0.814	0.825	Space–time allocation elasticity	0.672	0.571	Familiarity with digital platforms	0.678	0.706
~Penetration rate of health culture	0.449	0.453	~Space time allocation elasticity	0.585	0.503	~Familiarity with digital platforms	0.599	0.557
Level of shared responsibility	0.823	0.848	Depth of school community collaboration	0.717	0.603	Security Perception Index	0.823	0.799
~Level of shared responsibility	0.438	0.435	~Depth of school community collaboration	0.552	0.480	~Security Perception Index	0.425	0.422

“~” indicates logical “not”.

From the perspective of game theory, the analysis of necessary conditions does not identify any single antecedent that constitutes the necessary conditions for the sharing and opening of school sports resources, indicating that the result is not dominated by a single subject or single factor, but an equilibrium state formed under the interaction of multi-agent Strategic Interaction. In the game structure composed of social fitness population, school administrators and students, the three parties restrict each other in terms of resource requirements, risk-taking and rights protection. If either party adopts a positive or negative strategy alone, it is difficult to promote the stable opening of school sports venues without the cooperation of other subjects. Therefore, there is no single condition with absolute dominance for the sharing and opening of school sports resources, but depends on a variety of strategic combinations formed by different subjects in a specific situation, reflecting the Conditional substitution and Collaborative features in the multi-agent game.

### 4.2 Conditional configuration analysis

The opening of the school stadium to the society is a Governance process in which the Social fitness population, school administrators and students and other subjects participate. Its operation results show the realization of travel alienation in different situations. In order to identify the different implementation paths that affect the opening efficiency of school sports venues, this paper uses the fsQCA method to analyze the Configuration features of antecedents. In the specific analysis, set the case frequency threshold as 1, the consistency threshold as 0.8, and the PRI consistency threshold as 0.65 (0.8 for students) to ensure the robustness of the analysis results.

#### 4.2.1 Configuration analysis of social fitness population

In the game structure of school sports venues opening, the Social fitness population is not a simple resource user, but has an important impact on the trade-off between school opening scale and management risk through organizational participation, responsibility and behavior norms. Based on the Configuration analysis results of relevant conditions at the social level, the study identified three groups of antecedent condition configurations, with Overall consistency of 0.916 and Overall coverage of 0.709, indicating that social factors play a key role in the sharing efficiency of school sports resources through a variety of configuration paths in different situations (Table 4).

**Table 4:** Configuration analysis of Social fitness population.

Antecedent conditions	Resource sharing efficiency index		
	Configuration 1	Configuration 2	Configuration 3
Fitness demand intensity			
Diversity of demand		●	●
Digital divide coefficient		⊗	●
Degree of spontaneous organization	●	●	●
Penetration rate of health culture	●	●	
Level of shared responsibility	●		●
uniformity	0.924	0.945	0.959
Original coverage	0.663	0.351	0.437
Unique coverage	0.181	0.021	0.023
Overall coverage	0.709		
Overall consistency	0.916		

● indicates that the core is present, ● indicates that the edge is present, ⊗ indicates that the core is absent, ⊗ indicates that the edge condition is absent, and the blank space indicates that the condition has no effect on the result.

(1) Comparison of vertical features of configuration path

From the comparison results of the three Social configuration paths, different antecedents jointly shape the diversified realization mode of social forces’ participation in school sports resources sharing through Differentiated combination method. Path 1 shows the typical characteristics of “cultural responsibility of social self-organization”, which is the core path for society to promote the sharing of school sports resources. The core conditions of this path are Degree of spontaneous organization, Penetration rate of health culture and Level of shared responsibility. Its original coverage is 66.3%, and unique coverage is 18.1%, which is significantly higher than that of path 2 (35.1, 2.1%) and path 3 (43.7, 2.3%), indicating that this path has a higher explanatory power on resource sharing efficiency. Under the framework of game theory, this path corresponds to stable and balanced cooperation with social forces as the key fulcrum. The core decision of the school is to balance the scale of site opening and management risk, and the organizational participation of social subjects has changed this trade-off. Social self-organization reduces the cost of organization and implementation in resource opening by sharing coordination and maintenance affairs, so that the Open Strategy has higher expected benefits than the conservative strategy. The continuous penetration of health culture strengthens the value recognition of social members for resource sharing, stabilizes the expectation of expectations for school community cooperation, and reduces the cooperation risk caused by behavioral uncertainty. The Responsibility sharing mechanism alleviates the unilateral pressure of schools in the safety responsibility game by clarifying the boundaries of safety and management responsibilities. Under the synergistic effect of the above conditions, the income risk structure of the school has been changed, making its choice of continuous openness a rational and sustainable balanced result, and promoting the evolution of the path into a dominant path.

(2) Horizontal difference analysis of different configuration paths

From the horizontal comparison of different configuration paths at the social level, there are obvious differences in the role of antecedents in the formation of Resource sharing efficiency. “Degree of spontaneous organization” appears as the core condition in all configurations, which is the key antecedent of evaluating the opening efficiency of Opening up of school sports resources. The sharing of school sports resources does not rely on government instructions or one-way demand driven, but highly depends on the maturity of social self-organization ability. From the perspective of game theory, the existence of spontaneous organizations has changed the interactive structure between society and schools. By reducing information asymmetry and negotiation costs, the game that might have fallen into low-level cooperation has turned into a sustainable cooperative state. In this context, social organizations not only undertake the functions of coordination and mobilization, but also strengthen the rules and constraints through collective action to enhance the school’s expectations of trust in the external environment. “Fitness demand intensity” does not constitute a key condition in any path, reflecting that in a social environment with a high degree of organization and cultural identity, fitness behavior has changed from individual needs to stable social practice norms, and the focus of the game has shifted from demand strength to organizational ability and collaborative behavior.

#### 4.2.2 School administrators configuration analysis

In the process of opening the school stadium to the public, the school administrators are the main makers of resource allocation and operating rules, and their decision-making method directly affects the stability and sustainability of the site opening. Based on the Configuration analysis results of the relevant conditions of the school management, the study identified four representative precedent condition combinations from the school behavior pattern. The overall consistency is 0.857 and the overall coverage is 0.511, reflecting the practice path of promoting sports resource sharing through diversified management strategies in different governance situations (Table 5).

**Table 5:** Configuration analysis of school administrators.

Antecedent conditions	Resource sharing efficiency			
	Configuration 1	Configuration 2	Configuration 3	Configuration 4
Integrity of Open Policy		●	●	
System investment control	●	●	●	●
Policy compensation obtained	⊗	●	⊗	●
Maturity of risk management	⊗	⊗	●	●
Space–time allocation elasticity	⊗	●		●
Depth of school community collaboration	●		●	●
uniformity	0.931	0.932	0.926	0.851
Original coverage	0.281	0.240	0.269	0.396
Unique coverage	0.053	0.012	0.011	0.150
Overall coverage	0.511			
Overall consistency	0.857			

● indicates that the core is present, ● indicates that the edge is present, ⊗ indicates that the core is absent, ⊗ indicates that the edge condition is absent, and the blank space indicates that the condition has no effect on the result.

(1) Comparison of vertical features of configuration path

From the perspective of the configuration path at the school management level, the configuration mode of different governance elements directly shapes the logic of action and strategy choice of the school in the Open sports resources. As the core path, path 4 presents the typical characteristics of the “school-led Technology collaborative governance type”, which is the key implementation path for schools to promote sports resource sharing. The core conditions of this path are System investment control, Policy compensation obtained and Depth of school community collaboration, and Maturity of risk management and Space time allocation elasticity are auxiliary conditions. The original coverage is 39.6%, and the unique coverage is 15%, which is significantly higher than that of path 1 (28.1, 5.3%), path 2 (24.0, 1.2%) and path 3 (26.9, 1.1%), indicating that this path has a strong leading role in the formation of resource sharing efficiency. Combined with the analysis of game theory, this path corresponds to a Cooperative game equilibrium with schools as key actors and technology systems as coordination tools. Systematic investment and technical control have effectively alleviated the information asymmetry and coordination cost in the cooperation between schools and society, and enabled schools to obtain higher expected benefits in the trade-off between open and conservative strategies; the policy compensation mechanism improves the income structure of schools in the Open Resource Game and reduces their tendency to avoid costs and risks; the promotion of the depth of synergy between schools and society strengthens the foundation of trust in the repeated game, and guides all participants to continuously choose the Collaboration Strategy. Path 4: by synchronously optimizing the earnings expectation and risk sharing mechanism, the sustainable Open Strategy adopted by the school will become a stable and rational balanced result.

(2) Horizontal difference analysis of different configuration paths

From the perspective of the overall structure of each configuration path at the school level, the stability and replaceability of different conditions in the path show obvious differentiation. “System investment control” appears as a core condition in all configurations and is a key variable affecting the efficiency of school sports resources sharing. Schools’ promotion of resource opening does not rely solely on external policy pressure or willingness to cooperate, but reshapes the Game structure through institutionalized and technological system construction. System investment control with the help of Unified Resource Standards and the integration of facilities and staffing, the scattered sports venues and service elements are transformed into a schedulable and traceable sharing network, reducing the coordination friction between schools and society, and making resource sharing from loose collaboration to stable integration. At the same time, the role of “Maturity of risk management” in different paths is different, which only constitutes the core condition in some situations, indicating that when the System investment control level is high, the intelligent system has replaced the traditional risk management method to a certain extent, and realized Risk Identification and quick response through the real-time monitoring and early warning mechanism, so as to reduce the necessity of Artificial risk management in the game.

#### 4.2.3 Configuration analysis of students in school

In the context of opening school sports resources to the public, students, as direct users, exert a fundamental influence on the operational effectiveness of resource sharing through their participation behavior and risk perception, although they do not directly determine policy decisions (Question 3.2). The Configuration analysis around the relevant conditions at the student level found that the student behavior was mainly presented through the combination of four groups of precedent condition combination, with an overall consistency of 0.91 and an overall coverage of 0.487, indicating that the student factor formed a context-dependent mode of action on the sharing of school sports resources under different institutional arrangements and security expectations (Table 6).

**Table 6:** Configuration analysis of students in school.

Antecedent conditions	Resource sharing efficiency			
	Configuration 1	Configuration 2	Configuration 3	Configuration 4
Sports participation frequency				●
Site use rules	⊗		⊗	●
Rights appeal mechanism	●	●	●	●
Student autonomous organization	⊗	⊗		●
Familiarity with digital platforms		●	●	⊗
Security Perception Index	●	●	●	●
Uniformity	0.932	0.939	0.912	0.951
Original coverage	0.350	0.323	0.353	0.248
Unique coverage	0.061	0.028	0.039	0.031
Overall coverage	0.487			
Overall consistency	0.910			

● indicates that the core is present, ● indicates that the edge is present, ⊗ indicates that the core is absent, ⊗ indicates that the edge condition is absent, and the blank space indicates that the condition has no effect on the result.

(1) Comparison of vertical features of configuration path

Starting from the formation mechanism of students’ individual participation process, Path 1 reveals the basic logic of students’ participation behavior in the context of open resources (Question 3.2). Path 1 presents a typical “basic guarantee type of student participation”, which is an important way to promote the sharing of school sports resources at the student level. This path takes the Rights appeal mechanism and Security Perception Index as the core conditions. Its original coverage is 35%, and the unique coverage is 6.1%, which are higher than the other three paths, indicating that this path has high explanatory power in the formation of resource sharing efficiency. From the perspective of game theory, this path corresponds to a game structure with students as the key core and around “whether to enter the sharing process.” In this structure, when facing the opening of sports resources, students may exhibit different participation tendencies, while rights protection and safety expectations constitute the bottom-line constraints shaping these behavioral responses (Question 4). When the appeal channel is smooth and the safety perception level is high, the expected use income of students is significantly higher than the potential risk cost, and the participation behavior becomes the dominant strategy; if the basic rights and interests or safety expectations are difficult to be guaranteed, even if the school or society has a strong willingness to open up, students are more likely to take withdrawal or negative usage behavior. Therefore, path 1 provides a stable and sustainable starting point for the opening of school sports resources by consolidating the basic conditions for students’ decision-making.

(2) Horizontal difference analysis of different configuration paths

From the configuration results at the student level, whether students enter and continue to participate in Sports resource sharing first depends on their judgment of basic rights and interests and safety environment. The “rights appeal mechanism” and “Security Perception Index” appear in the form of core conditions in all configurations, which are the prerequisite and constraint for students to enter the resource sharing process. It shows that in the student-related Game structure, rights protection and security expectations are not subsidiary factors, but the key threshold to decide whether to choose the Collaboration Strategy. With the institutionalized Appeal channel, students can transform individual demands into responsive and adjustable governance needs, alleviate the problem of feedback lag in traditional management, and realize the dynamic adjustment of power and responsibility relationship. As the direct user of sports resources, students are highly sensitive to security risks. A higher safety perception level helps to reduce their psychological expected cost of potential risks and enhances their willingness to use. Accordingly, the “site use rules” do not constitute a core condition in any path, indicating that under the situation of effective operation of the appeal mechanism, students can flexibly modify the implementation of the rules through institutional channels, weakening the dependence on the preset rules, and shifting the focus of governance from static norms to rights protection.

### 4.3 Robustness test

The essence of fsQCA method is a set theory method, and there is a subset relationship between its results. If the operation steps are slightly adjusted and the research conclusion has not fundamentally changed, the results can be considered to be robust (51). The PRI consistency threshold was reduced from 0.8 to 0.75; the calibration anchor points of results and antecedents were adjusted to 0.75, 0.50 and 0.25 quantiles. The newly generated configuration has only slight changes, and the changes are not enough to support a completely different interpretation result, which proves that the research result is robust.

### 4.4 Route optimization measures

**Figure 2:**
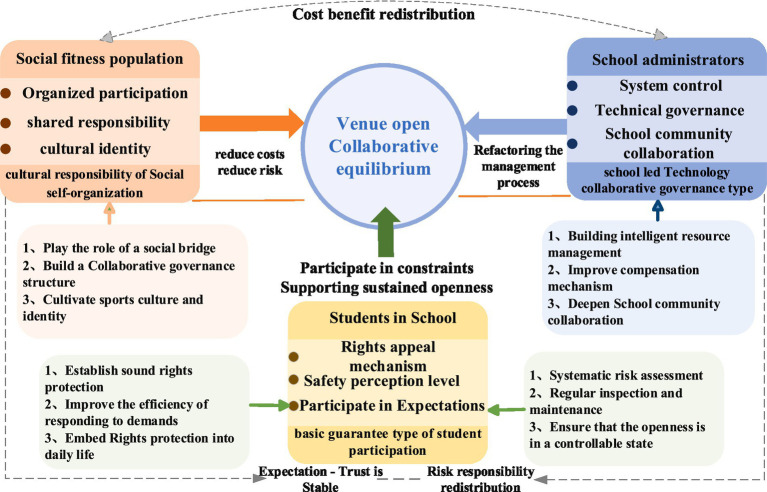
Collaborative governance through game theory mechanism.

From the perspective of game theory, the opening of school sports venues involves the three main bodies of the Social fitness population, school administrators and students, and forms a continuous interaction around the right to use sports venues, safety responsibility and cost benefit distribution. The public focuses on improving the availability of sports resources. School administrators need to balance the cost of public services and safety. The willingness of students to participate and the level of safety perception will directly affect the difficulty of school order maintenance and management, thus forming constraints on school-related decisions. The combination of different subject conditions determines whether the opening of school sports venues can achieve a sustainable cooperative equilibrium. Based on the three types of typical configuration path identified by fsQCA, the corresponding governance implementation directions are proposed from the three levels of Social fitness population, School administrators and students (Figure 2).

(1) In the process of opening school sports venues, the intervention of social forces directly affects whether the cooperation between schools and society can form stable expectations. The path of “cultural responsibility of social self-organization” shows that the organizational participation and responsibility embedding of social subjects can effectively alleviate the cost and risk pressure of schools in the opening of resources, so as to promote multiple subjects to form a cooperative relationship around public interests. ① Through social organizations to play the role of bridge, promote the exchange of sports facilities and professional resources between schools and communities, and change the situation that schools bear the cost unilaterally in the Open Resource Game. ② Build a collaborative governance structure characterized by government guidance, social coordination and school participation, so that different subjects can form relatively stable earnings expectations and division of responsibility in the process of resource opening, and reduce the uncertainty of the game. ③ Cultivate Shared Value Consensus through continuous Sports Culture Interaction, transform the opening of Open sports resources from the external system into the conscious action of social members, and reduce the possibility of cooperation rupture caused by the adjustment of subject strategies. The collaborative promotion of the above mechanisms is conducive to solidifying the opening of school sports resources into a long-term stable collaborative equilibrium state.

(2) Compared with the Social fitness population and students in school, school administrators play the dual roles of resource controller and governance coordinator in the opening of sports venues. The choice of governance tools directly determines the operation efficiency of the opening mechanism. The “school-led Technology collaborative governance type” path shows that the reconstruction of management processes with technology as the core is an important measure to enhance the synergy between schools and society. ① Build an Intelligent Resource Management Center, build a Unified Management System covering the school and surrounding communities, monitor the use status of sports venues in real time, and implement dynamic allocation according to different periods of time and space requirements to reduce idle and excessive use of resources. ② Embed policy compensation and collaboration rules into the technical system, transform the cooperative behavior between schools and society into an executable institutional process, stabilize the earnings expectations of all participants, and enhance the sustainability of cooperation. ③ Relying on the technology system, we should deepen the coordination between schools and society, so that schools can continue to maintain the coordination advantage in the Open Resource Game, and promote the evolution of sports resource sharing from phased cooperation to long-term stable coordination and equilibrium.

(3) In the process of opening school sports resources to society, whether students are willing to enter and continue to participate is an important basic condition affecting the effect of opening. The “basic guarantee path of students’ participation” shows that only under the premise that rights protection and safety expectations are systematically responded to, can students’ participation behavior occur stably. ① We will improve the rights protection system and form an efficient appeal processing closed loop. By building a Linkage Appeal Network between classes and schools, and combining online and offline channels, we can improve the transparency and processing efficiency of appeal response, and embed Rights protection into the daily operation of resource sharing. ② Improve the security prevention and control arrangements and reduce the risk expectation of students entering the sharing process. Around the facilities, behaviors and personnel security, establish a systematic risk assessment and Emergency management mechanism, and implement Regular inspection and dynamic maintenance for key sites and facilities to ensure that the opening of resources is under control. The linkage implementation of the above institutional arrangements and safety measures helps to stabilize the use expectations of students, so as to provide continuous and reliable support for the sharing of school sports resources.

## 5 Discussion

The findings further suggest that the opening of school sports resources operates within a multi-agent interaction framework rather than a linear policy implementation process. The absence of necessary conditions suggests that resource sharing efficiency is not determined by any single factor, but by the coordinated interaction of multiple conditions across different dimensions. From the perspective of game theory, this reflects a situation in which actors operate under mutual constraints, and the final outcome emerges as an equilibrium shaped by interdependent strategic choices. This provides a theoretical explanation for why different configurations can lead to similar levels of resource sharing efficiency.

At the structural level, the results further demonstrate that each actor promotes resource sharing through distinct yet interrelated mechanisms. From the social perspective, organized participation and normative consensus reduce uncertainty and stabilize cooperation, thereby creating favorable external conditions for sustained interaction. From the school perspective, institutional arrangements and technological governance reshape the balance between risks and benefits, enabling schools to shift from risk-avoidance strategies to cooperative strategies. From the student perspective, participation behavior and security perception function as key constraints on governance effectiveness, influencing whether resource sharing can be smoothly implemented in practice. These dimensions do not operate independently, but jointly shape the governance conditions under which resource sharing becomes sustainable.

More broadly, the findings highlight the configurational nature of school sports resource sharing. The coexistence of multiple effective pathways reflects the principle of equifinality, indicating that no single governance model can be universally applied. Instead, governance strategies should be adapted to specific institutional and social contexts, taking into account variations in social organization, school capacity, and user participation conditions. The robustness of the results further confirms that these patterns are not sensitive to model specifications, suggesting that they represent relatively stable structural relationships. Therefore, this study contributes to a more comprehensive understanding of how multi-agent interaction and configurational conditions jointly shape the efficiency of resource sharing.

The research has some limitations. The analysis is based on cross-sectional data, so it is difficult to completely exclude the reverse causality. Although fsQCA effectively identifies the configuration relationship between multiple conditions, it does not explicitly deal with the time sequence or causal direction; therefore, these findings should be interpreted as configuration correlation rather than strict causal inference. In addition, this study focuses on the configuration at the structural level, does not examine the mechanism at the micro level, and the sample is limited to specific regions and stakeholder groups, which may affect universality. Future research can better capture the dynamic process by incorporating longitudinal or panel data, use qualitative case studies to explore potential mechanisms, and expand the scope to include cross regional and different institutional backgrounds, so as to improve external effectiveness and solve these limitations.

## 6 Conclusion

From the perspective of a multi-agent game, this study discusses the opening of school sports resources and uses the fsQCA method to explore the allocation mechanism of resource sharing efficiency. The results show that: First, there is no prerequisite for achieving high resource sharing efficiency; on the contrary, the outcome is shaped by the joint action of multiple interactive factors.

Second, three representative allocation approaches are identified: (1) The typical feature of “cultural responsibility of Social self-organization” is the core path for the society to promote the sharing of School sports resources; (2) the characteristic of “school led Technology collaborative governance type” is the key implementation path to promote Sports resource sharing at the school level; (3) the typical feature of “basic guarantee type of student participation” is an important way to promote the sharing of School sports resources at the student level.

Third, resource sharing demonstrates strong environmental dependence and dynamic adaptability, indicating that governance strategies should be aligned with specific institutional and social conditions rather than adopting a one-size-fits-all approach. At the same time, these findings also reflect that the effectiveness of resource sharing is closely related to the interaction among social participation, school governance, and student-related conditions, which jointly shape the formation of different outcomes under specific contexts. In addition, the analysis of the absence of high resource sharing efficiency shows that low efficiency tends to occur when several key conditions are simultaneously weak or absent. Specifically, at the social level, low levels of spontaneous organization and insufficient penetration of health culture weaken external support; at the school level, limited digital platform proficiency and immature risk management capacity constrain governance effectiveness; and at the student level, weak rights appeal mechanisms and low security perception further increase management costs and implementation risks. These conditions often appear in combination rather than isolation, jointly leading to low-efficiency outcomes, rather than representing a simple inverse of high-efficiency configurations (Question 6).

## Data Availability

The original contributions presented in the study are included in the article/supplementary material, further inquiries can be directed to the corresponding author.
